# A Novel Monoallelic Nonsense Mutation in the *NFKB2* Gene Does Not Cause a Clinical Manifestation

**DOI:** 10.3389/fgene.2019.00140

**Published:** 2019-02-26

**Authors:** Jerzy Kotlinowski, Karolina Bukowska-Strakova, Agnieszka Koppolu, Joanna Kosińska, Natalia Pydyn, Piotr Stawinski, Mateusz Wilamowski, Witold Nowak, Alicja Józkowicz, Jarosław Baran, Rafał Płoski, Jolanta Jura

**Affiliations:** ^1^Department of General Biochemistry, Faculty of Biochemistry, Biophysics and Biotechnology, Jagiellonian University, Kraków, Poland; ^2^Department of Clinical Immunology, Institute of Pediatrics, Jagiellonian University Medical College, Kraków, Poland; ^3^Department of Medical Genetics, Medical University of Warsaw, Warsaw, Poland; ^4^Postgraduate School of Molecular Medicine, Medical University of Warsaw, Warsaw, Poland; ^5^Department of Medical Biotechnology, Faculty of Biochemistry, Biophysics and Biotechnology, Jagiellonian University, Kraków, Poland

**Keywords:** NF-κB signaling, *NFKB2* gene, nonsense mutation, common variable immunodeficiency, whole exome sequencing

## Abstract

NF-κB signaling, acting through *NFKB1* dependent canonical and *NFKB2* dependent non-canonical pathways plays a critical role in inflammatory and immune responses. Recent studies have associated mutations in these two genes with a common variable immunodeficiency (CVID). While evaluating a female patient seeking a diagnosis explaining her recurrent infections, we found a novel heterozygous c.1831C > T (p.Arg611^∗^) nonsense mutation in the *NFKB2* gene which introduces a Stop codon in the ankyrin repeat domain of p100. Whole exome sequencing (WES) analysis, followed by Sanger sequencing, identified this previously unknown mutation in two other family members. Penetrance of the c.1831C > T variant was assessed by flow-cytometry and protein expression in peripheral blood mononuclear cells (PBMC); whereas, activation of the NF-κB2 signaling pathway was examined through immunoblotting and real-time PCR. Heterozygous c.1831C > T variant led to the expansion of lymphocyte B subpopulations with concomitant reduction of plasmablasts, low IgG levels, and accumulation of p52 in PBMC. On the other hand, tested subjects had normal levels of IgM, IgA, IgE and no impairment in lymphocytes proliferation. Although evaluated patients did not fulfill all clinical features of CVID, their health should be monitored in the future for possible late manifestation of the disease. In conclusion, we showed that *NFKB2* haplodeficiency caused by c.1831C > T nonsense mutation is asymptomatic, possibly due to the compensatory mechanisms and allele redundancy.

## Introduction

**FIGURE 1:**
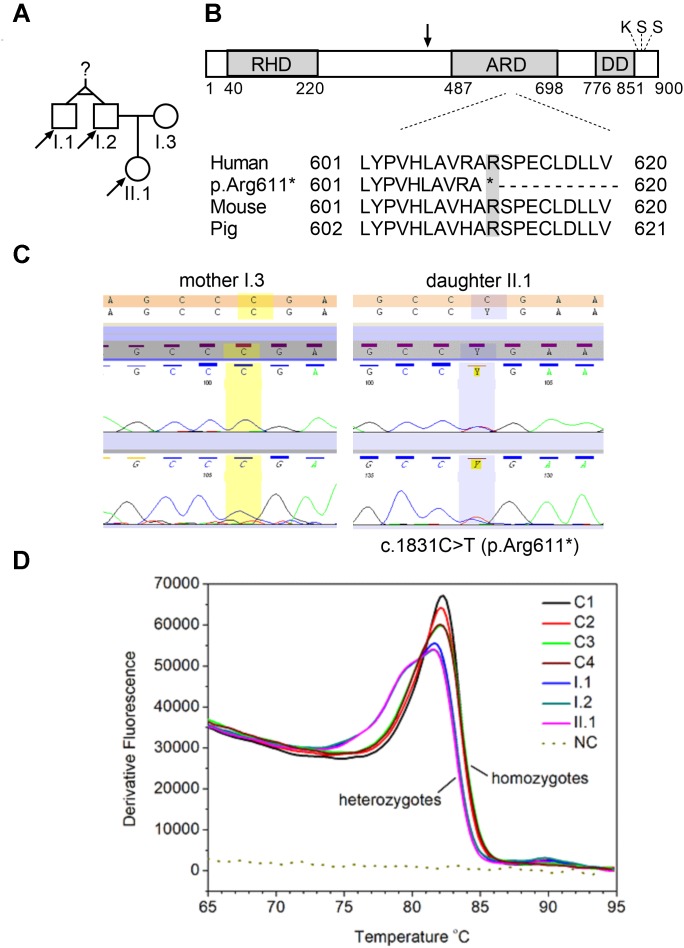
*NFKB2* c.1831C > T nonsense mutation. **(A)** Pedigrees of tested family, arrows indicate family members diagnosed with c.1831C > T (p.Arg611^∗^) nonsense mutation that were seeking a genetic testing. **(B)** Schematic representation of p100 domains showing rel homology domain (RHD), ankyrin repeat domain (ARD), and death domain (DD). Black arrow indicate processing position of p100, the location of the conserved lysine (K855) and two conserved serine s (S866 and S870) is also depicted on the scheme (Wietek and O’Neill, 2007, modified). Multiple sequence alignment of amino acid sequences in the fragment of ARD domain. **(C)** Sanger sequencing of two individuals over the c.1831 position. Left panel shows wild-type c.1831 position (mother, I.3) and right panel shows the c.1831C > T variant (daughter, II.1). **(D)** High resolution melt analysis of DNA product amplified in the real-time PCR reaction. 49 bp amplicons generated over mutated nucleotide were analyzed by HRM. Only one product is detected in control subjects, whereas all three samples collected from *NFKB2* haplodeficient subjects generated a bimodal melt curve, together with a shift toward lower melting temperature.

The human *NFKB2* gene locus (chromosome 10q24) encodes a p100/p52 transcription factor that belongs to the NF-κB signal transduction pathway. In mammals, this family consists of five members: p65 (RelA), RelB, c-Rel, NF-κB1 (p105/p50), and NF-κB2 (p100/p52). The canonical pathway, which includes NF-κB1, mediates a broad spectrum of inflammatory responses; whereas, B-cell survival and maturation, lymphoid organogenesis, dendritic cell activation, and bone metabolism are regulated by the non-canonical NF-κB2 pathway (Hayden and Ghosh, 2011; Sun, 2012). In the non-activated resting state, homo- and heterodimer of NF-κB proteins are retained in the cytoplasm by their association with inhibitory IκB proteins or by interaction with the C-terminal Iκ-homologous domain within their structure. Thus, full-length NF-κB1 (p105) and NF-κB2 (p100) proteins act as their own inhibitors (Figure 1C). For these proteins, proteasomal processing is required before translocation to the nucleus, where NF-κB1 (p50) and NF-κB2 (p52) bind to their target genes. Activation of NF-κB2 is triggered by signaling from a subset of TNFR members leading to NF-κB inducing kinase (NIK) accumulation in the cytoplasm. NIK triggers a kinase leading to phosphorylation of p100 at two conserved C-terminal serines (Ser866, Ser870) by IKKα kinase. This is followed by ubiquitination of lysine 855 and subsequent proteasomal processing, removing C-terminus from p100 to generate p52. Heterodimer of p52 and RelB is then translocated into the nucleus where this active complex acts as a transcription factor (Oeckinghaus et al., 2011).

Common variable immunodeficiency (CVID) is one of the most common primary immunodeficiencies, occurring in approximately 1:10,000 to 1:50,000 people. CVID is a clinically and genetically heterogeneous disorder characterized by recurrent infections, antibodies deficiency, defects in B-cell differentiation, and T cell abnormalities (Bonilla et al., 2016). Genetic defects responsible for CVID have been identified in less than 10–15% of all cases and include mutations in genes involved in lymphoid organogenesis and B-cell survival and maturation (Kienzler et al., 2017). Among them, there are also genetically defined patients with CVID and mutations in the *NFKB1* and *NFKB2* genes. To date, 9 mutations in *NFKB2* have been recognized in patients diagnosed with CVID. Such patients were characterized by early-onset CVID associated with autoimmunity, reduction in circulating B cells, adrenocorticotropic hormone deficiency, and occasional other pituitary hormone deficiencies. Interestingly, all of these reported mutations alter the amino acid sequence near the C-terminus of p100, a region crucial for NIK mediated p100 processing. As a result, p100 phosphorylation is blocked, inhibiting processing into the -52 active form and preventing nuclear translocation (Chen et al., 2013; Brue et al., 2014; Lee et al., 2014; Lindsley et al., 2014; Liu et al., 2014; Lougaris et al., 2015; Shi et al., 2016). This pathophysiological mechanism is mimicked in *Nfkb2* Lym1 mutated mice containing a non-processable form of p100 protein due to the p.Tyr868^∗^ nonsense mutation (Tucker et al., 2016). In addition, Kuehn and coworkers recently found two new heterozygous NFKB2 mutations (p.Glu418^∗^ and pArg635^∗^) resulting in constitutive p100/p52 activation, nuclear localization and gene transcription (Kuehn et al., 2017). Interestingly, mutations were found in both the asymptomatic subjects and patients suffering from immunodeficiency(Kuehn et al., 2017).

In our study, while evaluating a female patient suffering from recurrent infections and her relatives, we found a novel c.1831C > T (p.Arg611^∗^) nonsense mutation in the *NFKB2* gene that introduces a Stop codon in the ankyrin repeat domain (ARD) of p100. This mutation resulted in the expansion of lymphocyte B subpopulations with concomitant reduction of plasmablasts, low levels of IgG, and accumulation of p52 in peripheral blood mononuclear cells (PBMC).

## Results

### Genetic Analysis and Exome Sequencing

**FIGURE 2:**
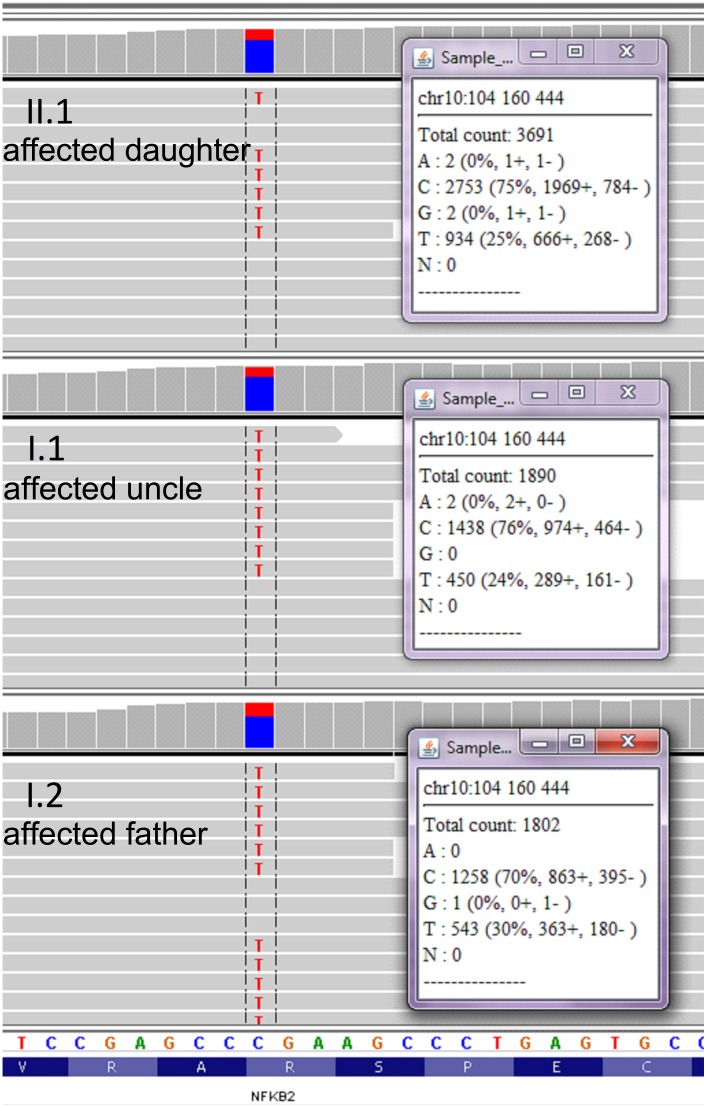
Analysis of wild type and mutated NFKB2 transcripts. RNA isolated from leukocytes was used for cDNA synthesis. Sanger sequencing was performed on cDNA product generated after PCR over the c.1831 position. Wild-type and mutated transcripts were detected in all individuals characterized by c.1831C > T mutation.

We collected a venous blood sample from a 28-year old female patient who was seeking a diagnosis explaining her recurrent infections and general ill health of unknown cause. The family history was insignificant apart from a history of a poorly described chronic childhood disease in her paternal uncle which was treated with IV IgG. Together with the Proband sample (II.1), we collected blood from three other family members, namely: father (I.2), his monozygotic twin brother (the paternal uncle, I.1) and mother (I.3) (Figure 1A). Genomic DNA was isolated after each subject provided written informed consent, in agreement with the Bioethics Committee at the Jagiellonian University in Krakow. Whole exome sequencing (WES) was performed to the mean depth of 45×, 77% of target was covered min. 20×, 89% – min. 10×. After variant filtration by exclusion of those reported as common (minor allele frequency >0.01), we focused on homozygous or potentially compound heterozygous variants (compatible with recessive inheritance) and ultrarare monoallelic variants predicted to cause a loss/decrease of function (Supplementary Table S1). Our approach led to the prioritization of a novel nonsense mutation in the coding exon (17th exon consisting of 169 nucleotides) of the *NFKB2* gene, namely chr10:104160444, C > T NM_001077494.3:p.Arg611^∗^/c.1831C > T. This monoallelic mutation was identified in the DNA isolated from individuals I.1; I.2, and II.1 but not I.3 (Figure 1A and Supplementary Figure S1) and was confirmed by Sanger sequencing (Figure 1B,C). The mutation was absent in the GnomAD database^1^ as well as from our in house database of >1000 Polish exomes. The c.1831C > T nonsense mutation, as described by us, is localized in the ARD domain of the p100 protein but it was unknown whether the mutated allele is expressed and whether this leads to translation of the truncated protein (Figure 1). To verify the presence of mutated mRNA in patients’ cells, we analyzed mRNA isolated from blood leukocytes. As shown in Figure 1D, a quantitative, high-resolution melt (HRM) analysis of a DNA fragment – a PCR product generated over a mutated nucleotide – indicated only one product in control patients. On the other hand, all three samples collected from *NFKB2* haplodeficient subjects generated a bimodal melt curve together with a shift toward lower melting temperature (Figure 1D and Supplementary Figure S2A). Additionally, Sanger sequencing of the same amplicon revealed that mutated transcripts represented 24–30% of all (Figure 2).

### Clinical Findings

Data from mouse models show that disrupted NF-κB2 signaling adversely affects both T and B cell function (Franzoso et al., 1998; Caamaño et al., 1998). Additionally, recently described mutations in humans leading to non-processable p100 were associated with the development of a common variable immunodeficiency (Chen et al., 2013; Lee et al., 2014; Lindsley et al., 2014; Liu et al., 2014). CVID in these patients was diagnosed at various ages and was linked to nonfunctional p100 protein. In contrast, according to medical records, only proband (II.1), and not two other subjects with c.1831C > T nonsense mutation, suffered from recurrent infections. Analysis of peripheral blood samples from proband’s father (I.2) and uncle (I.1) (subject II.1 was no longer available for blood analysis) revealed no abnormalities in lymphocytes subpopulations – total lymphocyte count, lymphocytes T and B, and NK cells were all within normal range (Table 1). We detected only a slightly decreased number of red blood cells in both individuals 4.34 and 4.24 mln/μL (normal range 4.30–5.80 mln/μL) in I.1 and I.2, respectively (Supplementary Table S2). Also, both men had normal numbers of circulating CD4+ and CD8+ T cells (Supplementary Table S3). Immunophenotyping of naïve, central memory, effector memory, and TEMRA cells also revealed no abnormalities (Supplementary Table S3).

**Table 1:** Leukocyte classes in subjects with mutation in *NFKB2* gene.

Subject	I.1	I.2	Normal range
**Lymphocyte subpopulations**			
Total lymphocyte count (cells/μL)	1892	2160	1000–3400
lymphocytes (% of WBC)	35.7%	40%	16.0–44.0%
CD3+ (cells/μL)	1470	1348	700–2100
CD4+ (cells/μL)	713	795	300–1400
CD8+ (cells/μL)	630	497	200–900
CD19+ (B cells, cells/μL)	216	177	100–500
CD3-CD16+CD56+ (NK cells, cells/μL)	210	**626**	90–600
CD3-CD16+CD56+ (NK cells, % of lymphocytes)	11.1%	29%	7–31%


*Normal values were taken from the EUROclass trial published by Wehr et al. (2008). Abnormal values are highlighted in bold.*

Further analysis of lymphocyte B subpopulations was performed according to EUROclass classification (Wehr et al., 2008). The circulating B-cell pool consists of up to 6 distinct subpopulations (Table 2) and the distribution of these subpopulations reflects differentiation of B cells in primary and secondary lymphoid tissue. In both subjects, we detected normal B-cell numbers (216 and 177 cells/μL, normal range 100–500, 11.4 and 8.2% of lymphocytes, normal range 6.0–19.0%), and normal percentage of class-switched memory B cells (10.47 and 9.69% of B cells, normal range 6.5–29.20%). However, four B-cell subpopulations were expanded: memory B cells (7.11 and 5.26% of lymphocytes, normal range 0.56–1.76%), marginal zone B cells (50.77 and 52.7% of B cells, normal range 7.2–30.80%), activated B cells (13.09 and 16.86% of B cells, normal range 1.10–6.90%), and transitional B cells (10.85 and 7.35% of B cells, normal range 0.60–3.50%) (Table 2). Of note is that expansion of selected B-cell subclasses was not observed among plasmablasts. In fact, we detected low numbers of these cells (0.2 and 0.18% of B cells, normal range 0.40–3.60%) as well as hypogammaglobulinemia (6.19 and 6.46 g/L, normal range 7–16 g/L). Although immunoglobulin levels are usually reduced in patients with CVID, we detected normal amounts of IgA, IgM, IgE, and complement proteins (C3c, C4) (Table 2). In the last set of experiments, we measured lymphocyte proliferation in response to phytohemagglutinin (PHA), pokeweed mitogen (PWM), and anti-T3 antigen antibodies, knowing that this *in vitro* test provides a semiquantitative assessment of total cell-mediated immunity. As shown in Table 2, stimulation of cells with either PHA, PWM or anti-T3 antibodies did not show any diminished proliferative responses (Table 2).

**Table 2:** Laboratory findings in subjects with mutation in *NFKB2* gene.

Lymphocyte B subpopulations	I.1	I.2	Normal range
B cells: CD19+ (cells/μL)	216	177	100–500
B cells (% of lymphocytes)	11.4	8.2	6.0–19.0%
Memory B cells: CD19+CD27+ (% of lymphocytes)	**7.11%**	**5.26%**	0.56–1.76%^∗^
Marginal zone B cells: CD19+CD27+IgD+ (% of CD19+)	**50.77%**	**52.7%**	7.20–30.80%
Class-switched memory B cells: CD19+CD27+IgD-IgM- (% of CD19+)	10.47%	9.69%	6.50–29.20%
Activated B cells: CD21lowCD38lowCD19high (% of CD19+)	**13.09%**	**16.86%**	1.60–10.00%
Total transitional B cells: CD38++IgMhigh (% of CD19+)	**10.85%**	**7.35%**	0.60–3.50%
Plasmablasts: CD38++IgM-CD21lowCD19low (% of CD19+)	**0.2%**	**0.18%**	0.40–3.60%
**Immunoglobulins**			
IgG (g/L)	**6.19**	**6.46**	7.00–16.00
IgA (g/L)	1.50	1.73	0.67–3.67
IgM (g/L)	0.68	1.06	0.41–2.30
IgE (IU/mL)	<4.63	14.80	0.00–100.00
**Complement**			
C3c (g/L)	0.90	0.98	0.75–1.36
C4 (g/L)	0.14	0.16	0.13–0.40
**Lymphocyte proliferation assay**			
Phytohemagglutinin (stimulation index)	69	87	>10
Anti-T3 antigen antibodies (stimulation index)	79	89	>10
Pokeweed mitogen (stimulation index)	74	105	>10


*An asterix indicates normal values taken from the Freiburg classification (Warnatz et al., 2002), with other normal values taken from the EUROclass trial published by Wehr et al. (2008). Abnormal values are highlighted in bold.*

**FIGURE 3:**
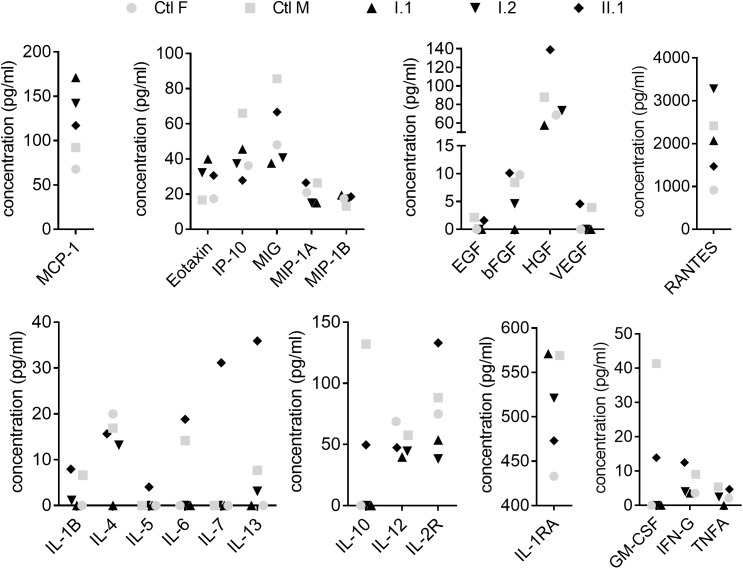
Plasma analysis. Plasma concentration of cytokines, chemokines and growth factors were measured by Human Cytokine Magnetic 30-Plex Panel. Filled symbols indicate family members (I.1, I.2, II.1) diagnosed with c.1831C > T (p.Arg611^∗^) nonsense mutation. Concentrations of analytes in individuals were compared to age- and sex-matched controls (Ctl F, Ctl M).

Cytokines and chemokines play an important role in the orchestration of leukocyte biology and changes in their profiles were shown among CVID patients (Rezaei et al., 2008; Hel et al., 2014; Varzaneh et al., 2014). In our study, out of 30 analytes, the concentration of six (IL-2, IL-8, IL-15, IL-17, G-CSF, and IFN-A) was below the limit of detection and remaining concentrations were unchanged between subjects with the mutation and healthy controls (Figure 3).

### Expression of Mutant NF-κB2 Protein

**FIGURE 4:**
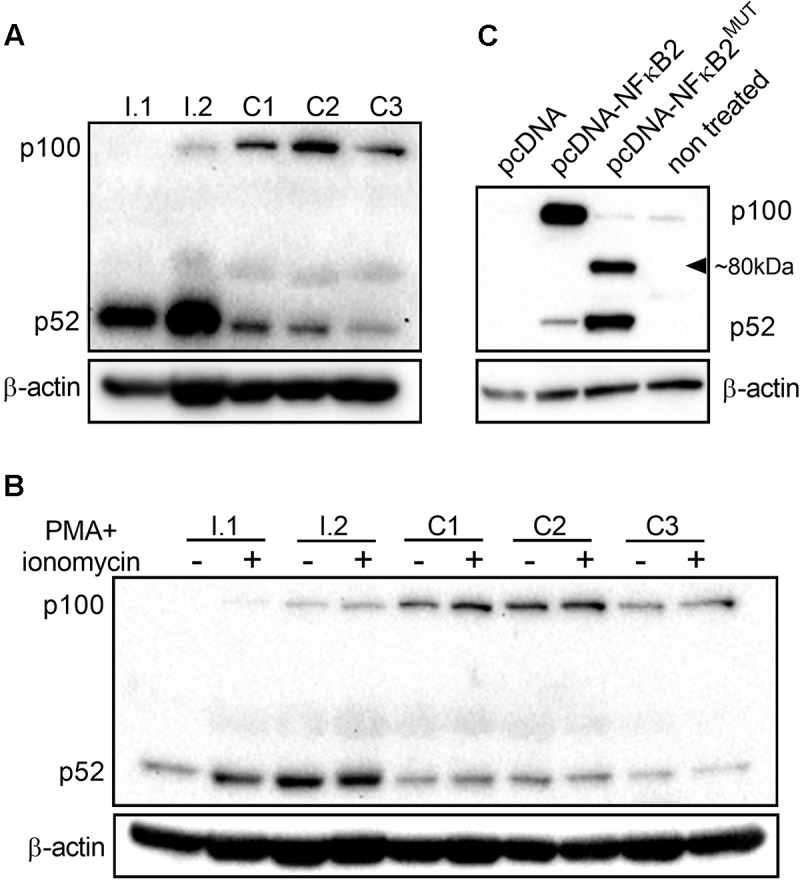
Expression of p100/p52 protein. **(A)** Immunoblot of whole-cell lysates from PBMC isolated from individuals with c.1831C > T mutation (I.1, I.2) and wild-type controls (C1, C2, C3). **(B)** Immunoblot of whole-cell lysates from freshly isolated PBMC and cells stimulated for 3 h with *phorbol* 12-myristate 13-acetate (PMA, 50 ng/mL) and ionomycin (1 μg/mL). Cells were isolated from family members (I.1, I.2) and wild-type controls (C1, C2, C3). **(C)** Before immunoblot analysis, HepG2 cells were transfected with expression vectors encoding coding sequence of wild-type *NFKB2* gene (pcDNA-NFKB2), or c.1831C > T mutated variant (pcDNA-NFKB2^MUT^).

*NFKB2* is a highly conserved gene and human p100/p52 shares 92% identity at the amino acid level with mouse protein. Since the c.1831C > T nonsense mutation identified by us might lead to generation of a truncated protein (610 amino acids instead of 900) deprived of regulatory residues Ser866, Ser 870, and Lys855, we hypothesized that the expression pattern of p100/p52 protein might be altered in individuals with the mutation. We assessed the level of p100/p52 proteins in peripheral blood mononuclear cells isolated both from family members with the mutation (I.1 and I.2) and unrelated control subjects (C1, C2, C3). Presence of the mutation was associated with decreased expression of the p100 subunit in PBMC but higher levels of p52 subunit as compared to age-matched controls (Figure 4A), which suggests an enhanced activation of the *NFKB2* gene in PBMC. Stimulation of PBMC for 3 h with Phorbol 12-myristate 13-acetate (PMA, 50 ng/mL) and ionomycin (1 μg/mL) did not change the expression pattern in mutation carriers; we detected low amounts of p100 and an accumulation of p52 in comparison to the control PBMC (Figure 4B and Supplementary Figures S3A,B). Interestingly, we did not detect any band corresponding to the truncated form of protein consisting of 610 amino acids either in freshly isolated PBMC (Figure 4A) or in cells stimulated *in vitro* (Figure 4B). Next, to exclude a lineage-specific effect of the p.Arg611^∗^ mutation, we assessed the level of p100/p52 proteins in the HepG2 cell line (human hepatocellular carcinoma originated from endoderm) transiently overexpressing the *NFKB2* gene. We found very low levels of p100 and p52 in cells transfected with empty plasmid (pcDNA) or in the non-treated control (Figure 4C). Transfection with plasmid harboring CDS of the *NFKB2* gene (pcDNA-NFKB2) led to overexpression of both p100 and p52 proteins. In contrast, HepG2 cells transfected with plasmid encoding *NFKB2* CDS with point mutation (p.Arg611^∗^, pcDNA-NFKB2^MUT^) overexpressed p52 but not p100. Additionally, in these cells, we detected a band (at ∼80 kDa) corresponding to a truncated form of p100/p52 protein (Figure 4C). Higher expression of p52 in cells transfected with pcDNA-NFKB2^MUT^ in comparison to pcDNA-NFKB2 is in accordance with data obtained from PBMC. However, lack of truncated protein *in vivo* implies very low expression levels and/or a more complex mechanism of transcription and/or translation regulation.

**FIGURE 5:**
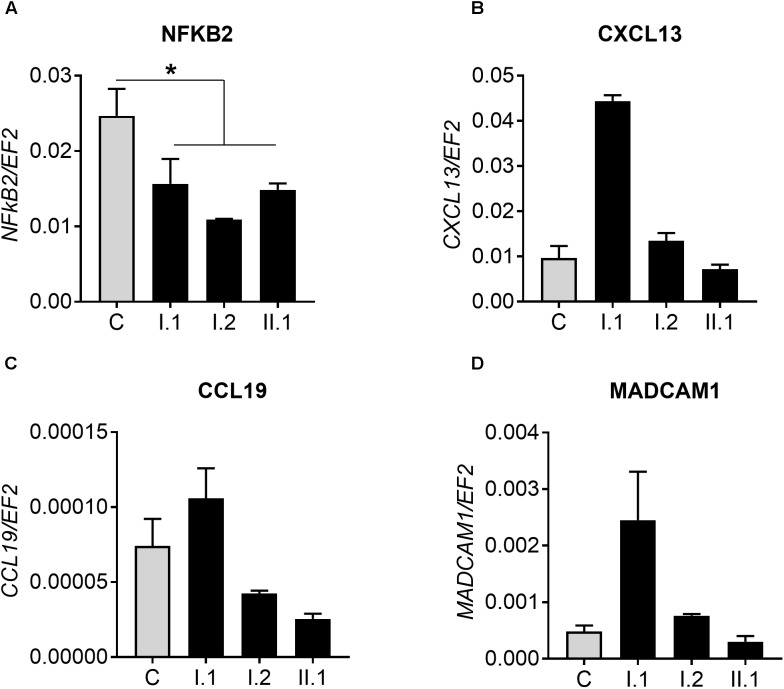
Real-time PCR analysis. **(A)** Expression of *NFKB2* gene in 10 controls **(C)** and analyzed subjects (I.1, I.2, II.2). Analysis of genes regulated by p100/p52 transcription factor: **(B)** *CXCL13*, **(C)** *CCL19*, and **(D)** *MADCAM1*. ^∗^*p* < 0.05.

To test for levels of non-canonical p100/p52 activation, we used real-time PCR to assay *CXCL13*, *CCL19*, and *MADCAM1*, known targets of the NFKB2 pathway. Firstly, we detected a reduced amount of *NFKB2* transcript in patients with p.Arg611^∗^ mutation and tested whether it influences expression of genes regulated by p100/p52 transcription factor (Figure 5A–D and Supplementary Figure S2B). However, there was no clear pattern of *CXCXL13* expression in subjects with mutation in comparison to controls: expression is higher (I.1), equal to (I.2) or lower than (II.1) in controls (Figure 5B). Similar results were obtained for two other genes; *CCL19* and *MADCAM1*, also regulated by p100/p52 transcription factor (Figure 5C,D).

## Discussion

In this study, we demonstrate that heterozygous c.1831C > T (p.Arg611^∗^) nonsense mutation in the *NFKB2* gene does not have an obvious clinical manifestation. Both analyzed patients do not suffer from severe infections, do not require repetitive immunoglobulin substitution therapy, and have no autoimmune manifestation. Laboratory analysis revealed that they fulfill only some criteria for CVID diagnosis. In 2008, after examination of 303 European CVID patients, a novel classification (EUROclass) of CVID subgroups based on detailed analysis of B lymphocytes was proposed (Wehr et al., 2008). Initially, EUROclass distinguishes between CVID patients with less or equal to 1% B cells (group B-) and those with more than 1% B cells (group B+). Group B+ patients included 90% with hypogammaglobulinemia that were enrolled in the study. Patients characterized by us, similarly to 29 subjects from the EUROclass trial, were classified as group B+smB+21^low^, based on the following criteria: more than 1% of B cells (B+), more than 2% of switched memory B cells (smB+), and expansion of CD21^low^ activated B cells. Importantly, the classification of CVID based on B-cell immunophenotyping cannot be the sole technique used for diagnosis. As emphasized in “International Consensus Document (ICON): Common Variable Immunodeficiency Disorders” published by a committee of immunology experts, patients should be diagnosed according to revised CVID criteria, taking into consideration the following aspects: (i) hypogammaglobulinemia must be accompanied by low levels of IgA or IgM; (ii) CVID patients should have an impaired response to either T-dependent or T-independent stimuli; (iii) differential diagnosis of hypogammaglobulinemia to exclude other diseases should be performed (Bonilla et al., 2016). Of note, most CVID patients will have at least 1 of the characteristic clinical manifestations (infection, autoimmunity, lymphoproliferation), however, the disease might also be diagnosed in asymptomatic individuals (Bonilla et al., 2016). Importantly, slightly different CVID criteria were proposed by other authors. For example, Ameratunga et al. (2014) suggested a cutoff <5 g/L for IgG concentration in all CVID patients, in contrast to the ICON document, wherein the authors recommend analysis based on regional clinical laboratory norms.

Study of consanguineous families with several members of CVID-like phenotype helped to identify genetic defects responsible for CVID development. Firstly, described mutated genes such as CD19, CD20, CD21, CD81, or ICOS affected B cell activation (Grimbacher et al., 2003; van Zelm et al., 2006, 2010; Warnatz et al., 2009; Kuijpers et al., 2010; Thiel et al., 2012). The list of mutations leading to CVID development is growing but genetic defects responsible for CVID still account for less than 10–15% of all cases (Salzer et al., 2012). Recent genome-wide association and whole genome sequencing studies have shown that whereas polygenic inheritance is frequent, a distinct monogenic cause of disease, including a defect in *NFKB2*, can be identified in a subset of CVID patients (Shi et al., 2016; Kienzler et al., 2017). To date, nine different mutations in the *NFKB2* gene, including point mutations, insertion, and deletions resulting in CVID development have been described. In all individuals characterized so far, NF-κB2/p100 processing and nuclear translocation were abrogated reflecting clinical manifestation of the disease (Chen et al., 2013; Brue et al., 2014; Lee et al., 2014; Lindsley et al., 2014; Liu et al., 2014; Lougaris et al., 2015; Shi et al., 2016). Whereas we cannot exclude that lack of penetrance in our family is caused by unidentified genomic variants, it is important to emphasize the *NFKB2* mutations described so far also change the amino acid sequence near the C-terminus of p100 and are associated with variable penetrance (Kuehn et al., 2017). The C terminus is crucial for NIK mediated p100 processing and lack of regulatory K855, S866 or S700 prevents the processing of the inhibitory precursor p100 into the active subunit p52. As a result, as described by Chen and coworkers, the level of p52 was reduced and p52 translocation to the nucleus was inhibited (Chen et al., 2013). In contrast to previously characterized mutations, Kuehn and coworkers identified two heterozygous nonsense mutations, p.Glu418^∗^ and pArg635^∗^, leading to constitutively active forms of p100/p52 associated with an immunodeficiency phenotype (Kuehn et al., 2017). One would expect, that changed amounts, or ratios of p100/p52 proteins would lead to altered gene expression. Indeed, besides well know non-canonical pathway, p100 was shown to sequester and inhibit NF-κB through formation of so called kappaBsomes (Tao et al., 2014). The unique ability of p100 to interact with all NF-κB subunits by forming kappaBsomes demonstrated its importance in regulation of cellular homeostasis by coordinating gene expression programs(Tao et al., 2014).

Clinical manifestation of p.Glu418^∗^ and pArg635^∗^ gain-of-function mutations included hypogammaglobulinemia, and recurrent upper and lower respiratory tract bacterial infections, all frequently described in CVID patients. However, the patients also developed problems not characteristic for CVID. Interestingly, both mutations were also detected in two asymptomatic carriers, thereby proving that immunological and clinical penetrance may not be complete (Kuehn et al., 2017). In line with this data, the p.Arg611^∗^ mutation identified by us causes no obvious clinical manifestation. However, both patients analyzed in detail were characterized by expansion of lymphocyte B subpopulations with concomitant reduction of plasmablasts and hypogammaglobulinemia, all characteristic for CVID. Although T cell abnormalities together with alternations in cytokine production have also been described in CVID patients, we did not detect any changes in these parameters (Varzaneh et al., 2014; Azizi et al., 2016, 2017). We also compared obtained results with recently published paper by Kachamakova-Trojanowska et al. (2018) who analyzed a cohort of 35 control samples by the same method. We found no difference in plasma concentrations of measured analytes between subjects with c.1831C > T mutation and control ones.

A lower amount of *NFKB2* transcripts in subjects with p.Arg611^∗^ prompts speculation that the nonsense mutation triggers elimination of mutant mRNA molecules by nonsense-mediated decay (NMD) resulting in a decreased pool of p100 transcript. As a consequence, p52 protein might be stabilized and accumulate in the PBMC. NMD is a surveillance pathway that reduces errors in gene expression by eliminating mRNA transcripts that contain premature Stop codons (Popp and Maquat, 2013). The best-understood mechanism of NMD action relies on pre-mRNA splicing and the presence of an exon junction protein complex (EJC) on the mature mRNA molecules. If during translation, the ribosome finds a Stop codon more than 50 nucleotides “upstream” of EJC, the cell recognizes mRNA as aberrant and destroys it (Popp and Maquat, 2013). In our subjects, c.1831C > T mutation introduces a Stop codon in the 17th exon, 137 nucleotides “upstream” of the exon junction complex and, according to current knowledge, should be assigned for NMD mediated degradation. In fact, sequencing of DNA product confirmed that mutated transcripts represent only between 24 and 30% of all *NFKB2* mRNAs. Such results might suggest either a lower transcription rate of mutated allele or a shorter half-life of mutated mRNA.

Lastly, expansion of four B-cell subpopulations in the analyzed subjects prompted us to check whether a nonsense mutation at a similar location in *NFKB2* was reported in lymphomas. Interestingly, in CVID patients, there is an enhanced incidence of several cancers, including lymphoma (Mortaz et al., 2016). However, there is no data on whether a specific genetic background might be the most prevalent cause of lymphoma in this group of patients. In fact, rearrangements of the *NFKB2* gene have been associated with both B-cell and T-cell malignancies. Such lymphomas are characterized by truncated p100 proteins that lack some of the C-terminal ankyrin repeats. A p100 mutant variant called p100HB originates from a nonsense mutation in the 21st exon of NFKB2, resulting in a protein lacking the last 125 amino acids. Protein p100HB was identified in several well-known human tumor cell lines derived both from B-cells (Daudi cell line) or T-cells (Jurkat cell line) (Derudder et al., 2003). HuT78 is another cell line originated from Cutaneous T cell lymphoma with mutated *NFKB2*. Kim and coworkers identified a protein consisting only of 666 amino acids (called p80HT) with the addition of a short (serine-alanine-serine) fusion at the 3′ end of p80HT. Interestingly, direct truncation at aa666 was fully inhibitory, as was a substitution of three alanines for the SAS residues. What is more, the presence of as few as two C-terminal ankyrin motifs in a protein consisting of 552 amino acids only was sufficient for inhibition of NF-κB-mediated transcriptional activation (Kim et al., 2000). Besides NFKB2 nonsense mutations detected in lymphomas, there is also a report of p.Arg609^∗^ mutation that was found in thyroid carcinoma. This mutation has been described in the COSMIC database and generates a protein shorter of just 2 amino acids than p.Arg611^∗^ (Catalogue of Somatic Mutations in Cancer, 2018). Although the actual C-terminal sequence of each of these proteins is distinct, the common C-terminal deletions suggest that this alteration may be important in changing the function of the NF-κB2 proteins.

In summary, we found a novel c.1831C > T (p.Arg611^∗^) nonsense mutation in the *NFKB2* gene, that introduces a Stop codon in ankyrin repeat domain (ARD) of p100. Various nonsense mutations in *NFKB2* gene were described to cause CVID, but patients evaluated by us were asymptomatic. They were characterized by expansion of lymphocyte B subpopulations with concomitant reduction of plasmablasts, low level of IgG and accumulation of p52 in peripheral blood mononuclear cells (PBMC) but did not fulfill other clinical features of CVID. Finally, in our opinion, the health status of c.1831C > T (p.Arg611^∗^) mutation carriers should be monitored in the future for possible late manifestation of the disease.

## Materials and Methods

### Subjects

Venous blood samples were collected from four family members: a 28-year old female patient (II.1), her uncle (I.1), father (I.2), mother (I.3) and unrelated control subjects. Informed consent for participation in the study and for the publication of this case report was obtained from all individuals and the study was approved by the Bioethics Committee at the Jagiellonian University in Krakow.

### Clinical Laboratory Studies

The levels of serum complement components C3 and C4 and total serum IgG, IgM and IgA and IgE concentrations were determined by nephelometry (Dade Behring/Siemens, Deerfield, IL, United States) using commercially available kits.

### *In vitro* Lymphocyte Proliferation Assay

Peripheral blood mononuclear cells were isolated from EDTA-treated peripheral blood by standard Ficoll density gradient. Isolated cells were resuspended in culture medium (RPMI-1640) supplemented with 10% fetal calf serum and antibiotics. Cells were cultured in microtiter plates (1 × 105 per well) at 37° with 5% CO2 for 72 h with following stimulants: phytohemagglutinin (PHA; 8 μg/mL), pokeweed mitogen (PWM; 2 μg/mL), or OKT3 (antibody to the T3 antigen of human T cells; 1 μg/mL). For the last 17 h of culture, the cells were pulsed with 1 μCi/well [3H]thymidine. Radioactivity of [3H]thymidine incorporated into cellular DNA was measured in a β scintillation counter and expressed as counts per minute (cpm). Stimulation index of lymphocyte proliferation was calculated as ratio of cpm signal after stimulation in comparison to unstimulated cells. Normal response to stimulants was considered at stimulation index greater than ten.

### Analysis of Peripheral Cell Subsets by Flow Cytometry

Enumeration of lymphocyte subsets was performed on EDTA-treated peripheral blood patients’ samples. In case of T cell subsets, peripheral blood cells were incubated with antibodies directly. In evaluating of B cell subsets, in purpose to detect surface immunoglobulis, blood samples were thoroughly washed in PBS prior to staining (to remove plasma immunoglobulins). The cells were stained for 15 min at room temperature with the following combinations of directly labeled monoclonal antibodies: (1) CD3-FITC/CD16+CD56-PE/CD45-PerCP/CD4-PE-Cy7CD19-APC/CD8-APC-H7 using BD Multitest 6-color TBNK reagent (total lymphocyte enumeration); (2) IgD-FITC/CD21-PE/CD45-PerCP/CD27-PE-Cy7/CD19-APC/CD38-AlexaFluor700/IgM-BV605 (B cell subsets enumeration); (3) CD3-FITC/CD25-PE/CD28Per-CP-Cy5.5/CD45RA-PE-Cy7/CD4-AlexaFluor700/CD8-APC-H7/CCR7-BV421/CD27-BC501/CD127-BV605 (T cell subsets enumeration). After incubation, erythrocytes were lysed using BD FACS Lysing Solution and washed twice in PBS (300 × *g*, 10 min, 4°C). Then, after two washing steps, the cells were resuspended in PBS and analyzed using a BD FACSCanto^TM^ 10-color flow cytometer (BD Biosciences) using BD FACSDiva v.8.01 software. The list mode data of 50,000 events in a “live gate” mode were acquired. The cells were gated on lymphocytes according to forward (FSC), side scatter (SSC) parameters and CD45 expression. In case of B cell subsets, results were given as the percentage of B cells and absolute counts per ml (due to EUROclass classifications recommendations). In case of T cell subsets results were presented as the percentage of lymphocytes and absolute numbers. Reference ranges of B cell subsets were adopted from EUROclass classifications.

### Whole Exome Sequencing (WES)

Whole exome sequencing was performed using HiSeq 1500 platform. Libraries were made using Nextera DNA Library Preparation Kit (Illumina). Bioinformatics analysis was performed as described previously (Ploski et al., 2014). *NFKB2* Sanger sequencing was performed to confirm genetic variants detected by WES.

Specific pair of primers: For 5′-ATGCCTGACTTTGAGGGAC-3′ and Rev 5′-ATGTCAGCACCAGCCTTCA-3′ was used to amplify a 299 bp fragment of *NFKB2* DNA. The amplified cDNA was processed using Nextera XT Library Preparation Kit (Illumina) and sequenced on HiSeq 1500.

### Immunoblotting

Cell lysates of PBMC and HepG cells were prepared in RIPA buffer with a Protease Inhibitor Cocktail (Roche) and a Phosphatase Inhibitor Cocktail (Sigma-Aldrich). Lysates were denaturated and subjected to SDS–PAGE electrophoresis. Blots were performed with the following antibodies: p100/p52 (Cell Signaling), β-actin (Sigma-Aldrich) and horseradish peroxidase-conjugated secondary antibodies (Sigma-Aldrich). Immunoblotting of β-actin was used as a loading control.

### Gene Expression

Total RNA was isolated from patients’ and controls’ leukocytes (after lysis of red blood cells) with a modified guanidinium isothiocyanate method. Next 1 μg of RNA was reverse transcribed into cDNA by M-MLV Reverse Transcriptase according to vendor’s protocol (Promega). Gene expression was measured by real-time PCR (Illumina) with the following specific primers: *NFKB2* For 5′-ATGCCTGACTTTGAGGGAC-3′ and Rev 5′-ATGTCAGCACCAGCCTTCA-3′; *CXCL13* For 5′-GGACCCTCAAGCTGAATGGA-3′ and Rev 5′-AGCTTGAGTTTGCCCCATCA-3′; *CCL19* For 5′-GGTGCCTGCTGTAGTGTTCA-3′ and Rev 5′-GCAGTCTCTGGATGATGCGT-3′; *MADCAM1* For 5′-GTGCTGTTCAGGGTGACAGA-3′ and Rev 5′-GTGCAGGACGGGGATGG-3′; *EF2* For 5′-GACATCACCAAGGGTGTGCAG-3′ and Rev 5′-TCAGCACACTGGCATAGAGGC-3′. The relative quantification of genes’ expression was calculated with the 2^-ΔCt^ method.

For sequence analysis on NFKB2 mRNA expressed in leukocytes we used cDNA synthetized as described above. Next, 299 bp DNA product was generated by PCR (with Phusion DNA polymerase with proof reading properties, Thermo Fisher) with the following primers: *NFKB2* For 5′-ATGCCTGACTTTGAGGGAC-3′ and Rev 5′-ATGTCAGCACCAGCCTTCA-3′. Finally, Sanger sequencing was performed to verify if mutated allele is expressed.

### High Resolution Melt Analysis

One microgram of total RNA isolated from leukocytes was reverse transcribed into cDNA by M-MLV Reverse Transcriptase according to vendor’s protocol (Promega). Next, a real-time PCR followed by high resolution melt (HRM) analysis was performed with the following primers: HRM_F: 5′-GTATCCAGTACACCTGGCGG-3′ and HRM_R: 5′-ATCCAGGCACTCAGGGCTTC-3′. After 40 cycles of standard real-time PCR reaction, 49 bp amplicons were analyzed by HRM according to the protocol: incubation at 60°C for 1 min followed by temperature change from 60 to 95°C with temperature ramp 0.1°C/s combined with signal detection.

### Cell Culture

HepG2 cells were cultured in DMEM LG with 10% fetal bovine serum and 2 mM L-glutamine at 37°C in a humidified 5% CO_2_ incubator. Cells were routinely tested for mycoplasma contamination by PCR.

### Plasmids Construction

The expressing vector coding for NFKB2 (pcDNA-NFKB2) was obtained by cloning of the PCR product to the mammalian expressing vector pcDNA3. Briefly, human coding sequence (CDS) of *NFKB2* gene was amplified on cDNA template obtained from PBMC isolated from healthy donor. Amplification was done using the following specific primers For 5′-CCAAGCTTTAGCCCAGAGACATGGAGAGT-3′ containing a HindIII restriction site at 5′ end and Rev 5′-ATGAATTCTTGAAATAGGTGGGGACGCTGTA-3′ containing an EcoRI restriction site at 5′end. After digestion of the PCR product with HindIII/EcoRI restriction enzymes the insert was cloned into pcDNA3 digested with the same enzymes as the insert. Generation of vector encoding a c.1831C > T nonsense mutation (p.Arg611^∗^, pcDNA-NFKB2^MUT^) was done by PCR site-directed mutagenesis with Phusion DNA Polymerase (Thermo Fisher Scientific) and the following primers: For 5′-TCCGAGCCTGAAGCCCTGAGT-3′ and Rev 5′-CCGCCAGGTGTACTGGATACA-3′. All generated vectors were sequenced, and their expression was confirmed by western blot with a p100/p52 specific antibodies.

### Luminex Analysis

Plasma concentrations of cytokines, chemokines and growth factors were analyzed by Luminex^®^ Technology (Human Cytokine Magnetic 30-Plex Panel, Life Technologies) in 96-well plates according to vendor’s protocol. Briefly, samples were mixed with buffer containing standards or specific antibodies bound to microspheres and incubated for 2 h at room temperature. Next, after buffer aspiration and washing with PBS, secondary antibodies conjugated with biotin were added. Directly before fluorescence measurement streptavidin-phycoerythrin complex was added to all samples.

### Statistical Analysis

Results are expressed as mean+SEM and were analyzed by GraphPad Prism Software (GraphPad). Two tailed Student’s *t*-test was used for comparison of two groups. The *p*-values are marked with the asterisks in the charts (^∗^*p* < 0.05) and differences were considered significant when *p* < 0.05.

## Author Contributions

JeK designed and performed the experiments, analyzed the data, and drafted the manuscript. KB-S, NP, MW, WN, AJ, and JB performed the experiments and critically reviewed the manuscript. AK, JoK, and PS performed the WES and Sanger sequencing studies. RP contributed with the patients to the study, undertook the DNA sequencing and bioinformatics analysis. JJ and RP conceived the study and wrote the manuscript.

## Conflict of Interest Statement

The authors declare that the research was conducted in the absence of any commercial or financial relationships that could be construed as a potential conflict of interest.

## Footnotes

1 http://gnomad.broadinstitute.org

## Supplementary Material

FIGURE S1 *NFKB2* sequencing. **(A)** Nonsense mutation c.1831C > T (p.Arg611^∗^) was found after whole exome sequencing analysis in proband’s DNA. **(B,C)** Presence of mutation was confirmed by Sanger sequencing in proband’s sample and tested in other family members.

FIGURE S2 Expression of mutated *NFKB2* allele. **(A)** Raw data of high resolution melt analysis. Real-time PCR was performed in triplicates and each curve corresponds to one sample (one well in the 96-well real-time PCR plate). Black curves represents control subjects, red curves represents subjects with c.1831C > T mutation. **(B)** Real-time PCR analysis of *NFKB2* expression in leukocytes collected from 10 control patients and 3 carriers of nonsense mutation c.1831C > T (p.Arg611^∗^).

FIGURE S3 Densitometry analysis. Amount of p100 **(A)** and p52 **(B)** proteins were evaluated by western blot and next quantified by densitometric analysis.
