# Supplementary figures and images for: A Novel Monoallelic Nonsense Mutation in the NFKB2 Gene Does Not Cause a Clinical Manifestation
Source: Front Genet. 2019 Feb 26;10:140. doi: 10.3389/fgene.2019.00140 (PMC6399389; doi:10.3389/fgene.2019.00140)

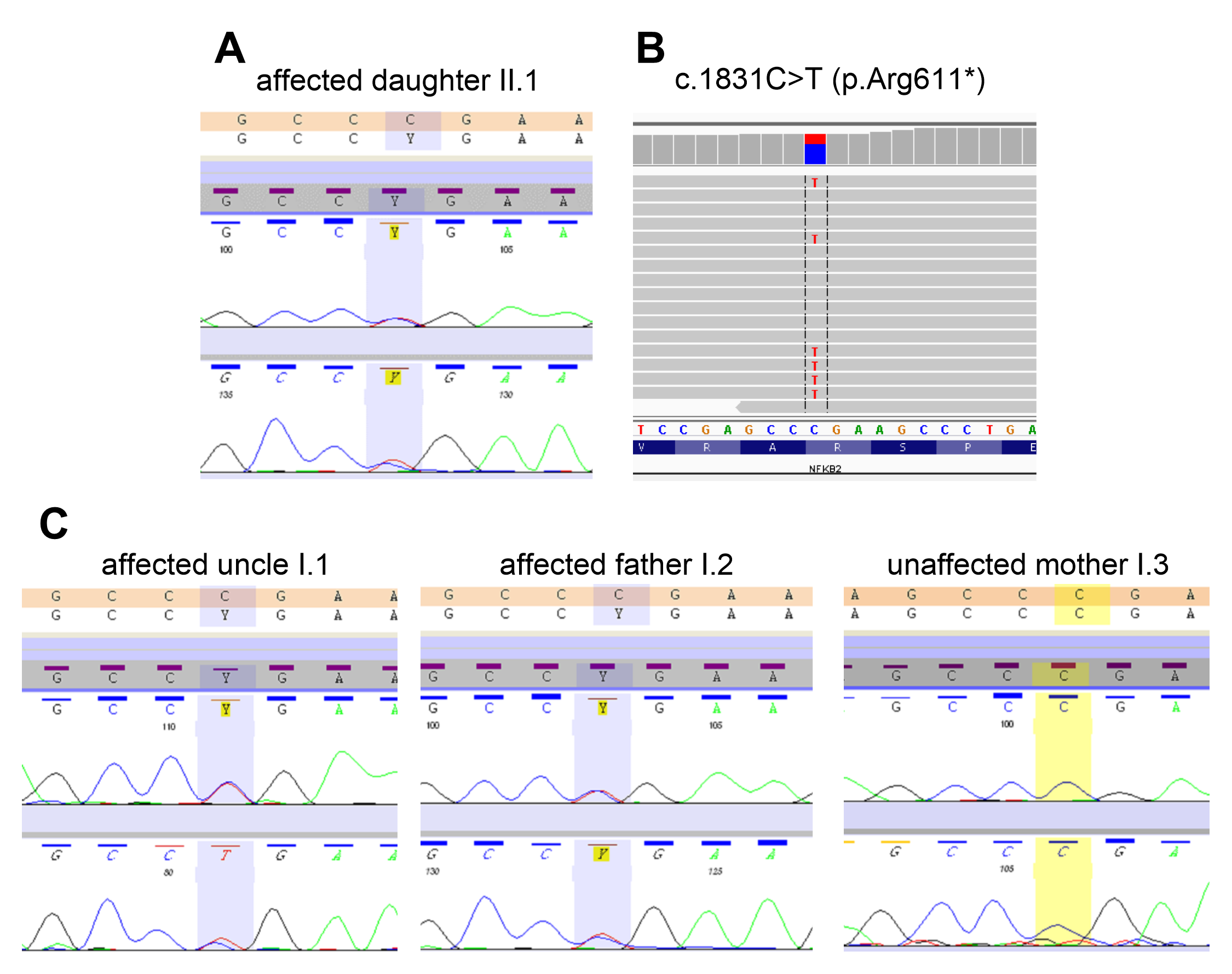

Supplement: FIGURE S1 — NFKB2 sequencing. (A) Nonsense mutation c.1831C > T (p.Arg611∗) was found after whole exome sequencing analysis in proband’s DNA. (B,C) Presence of mutation was confirmed by Sanger sequencing in proband’s sample and tested in other family members. [file Image_1.TIF]

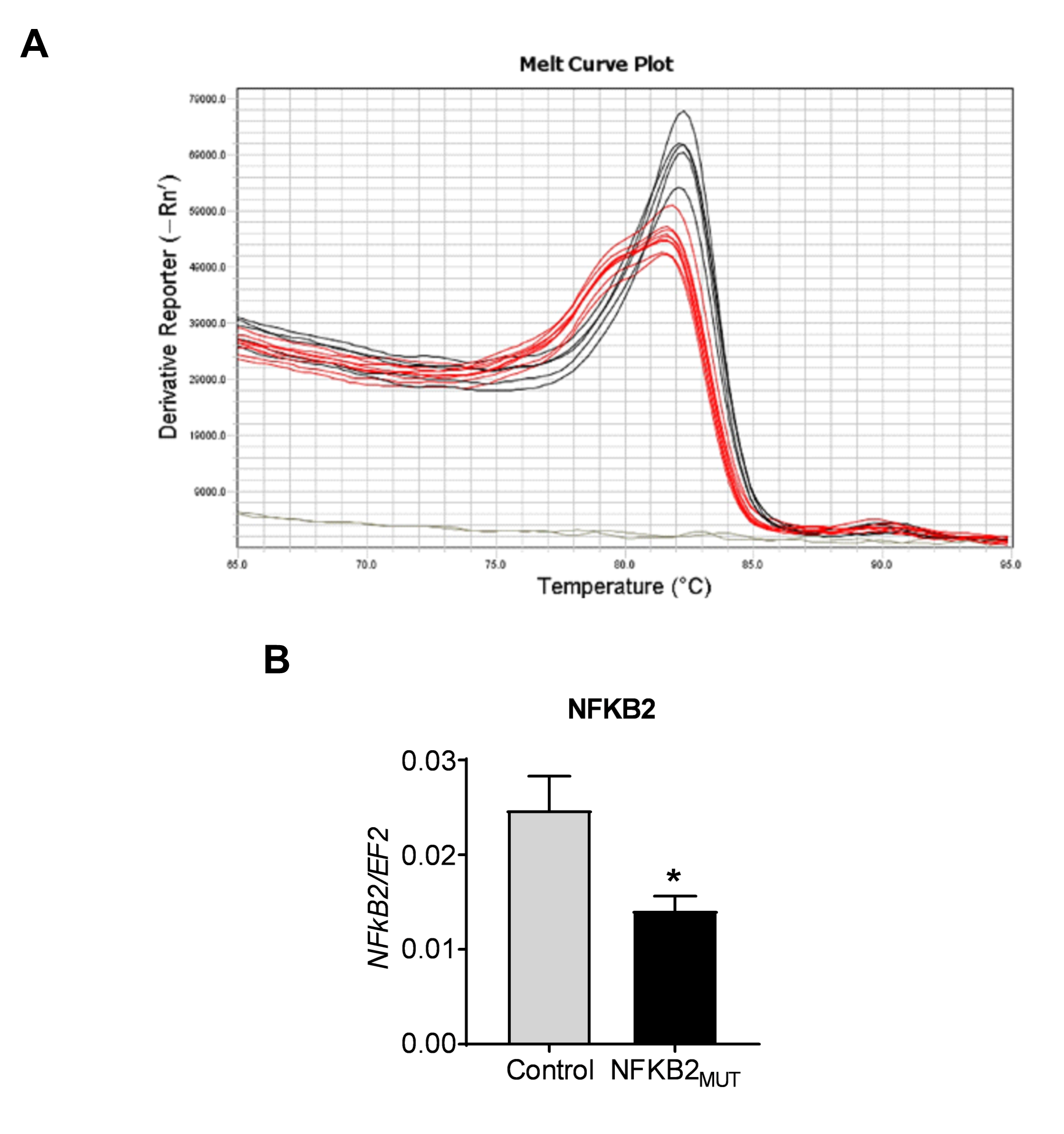

Supplement: FIGURE S2 — Expression of mutated NFKB2 allele. (A) Raw data of high resolution melt analysis. Real-time PCR was performed in triplicates and each curve corresponds to one sample (one well in the 96-well real-time PCR plate). Black curves represents control subjects, red curves represents subjects with c.1831C > T mutation. (B) Real-time PCR analysis of NFKB2 expression in leukocytes collected from 10 control patients and 3 carriers of nonsense mutation c.1831C > T (p.Arg611∗). [file Image_2.TIF]

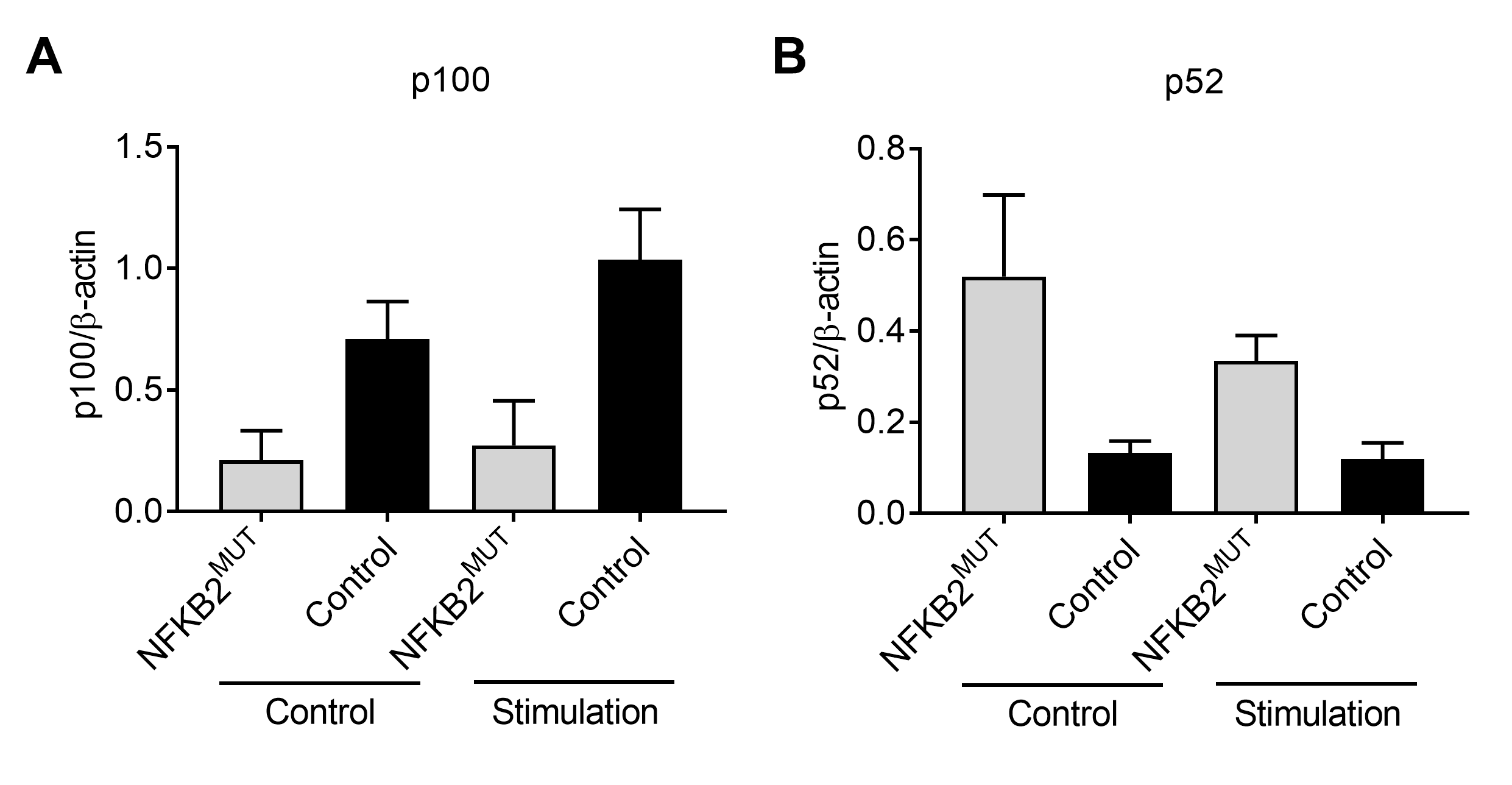

Supplement: FIGURE S3 — Densitometry analysis. Amount of p100 (A) and p52 (B) proteins were evaluated by western blot and next quantified by densitometric analysis. [file Image_3.TIF]
