# Supplementary material for: A Novel Monoallelic Nonsense Mutation in the NFKB2 Gene Does Not Cause a Clinical Manifestation
Source: Front Genet. 2019 Feb 26;10:140. doi: 10.3389/fgene.2019.00140 (PMC6399389; doi:10.3389/fgene.2019.00140)
Supplement: Supplementary file 4 [file Table_1.DOCX]

**Table S1. Variants considered as disease causing after whole exome sequencing in proband**

| **Gene** | **Position** | **ID** | **Effect** | **Disease (inheritance)** |
| --- | --- | --- | --- | --- |
| **Potentially autosomal recessive (AR, biallelic variants <0.01)** | | | | |
| *THOC1* | chr18:215504 | rs138671246 | NM_005131.2:p.Pro535Ser/c.1603C>T  (homozygous) | Not associated with human disease, variant not likely to be damaging (“tolerated” according to MetaSVM programme) |
| *DMXL2* | chr15:51773257 | rs146972389 | NM_015263.3:p.Asp2016Asn/c.6046G>A | Polyendocrine-polyneuropathy syndrome (MIM #616113), not consistent with symptoms |
| *DMXL2* | chr15:51768917 |  | NM_015263.3:c.6834G>A |  |
| *NBEAL1* | chr2:204013748 | rs199629983 | NM_001114132.1:p.Arg1751His/c.5252G>A |  |
| *NBEAL1* | chr2:203991290 | rs201546230 | NM_001114132.1:p.Thr970Ile/c.2909C>T | Not associated with human disease. All variants not likely to be damaging (“tolerated” according to MetaSVM programme) |
| *NBEAL1* | chr2:203972386 | rs746463904 | NM_001114132.1:p.Pro446Leu/c.1337C>T |  |
| **Potentially dominant (High effect, freq.=0 in available databases)** | | | | |
| *AGPAT9/GPAT3* | chr4:84518600 |  | NM_001256422.1:p.Thr310Ala/c.928A>G | Not associated with human disease. Altered energy and cholesterol homeostasis in knock-out mice. |
| *GABRA6* | chr5:161118998 |  | NM_000811.2:p.Ser293Phe/c.878C>T | Childhood Absence Epilepsy, Alcohol Dependence (MIM #103780), not consistent with symptoms |
| *RAPSN* | chr11:47459446 | rs886048385 | NM_005055.4:c.*80G>C,NM_032645.4:c.*80G>C | Myasthenic syndrome (MIM #616326), not consistent with symptoms |
| *PDE12* | chr3:57543021 |  | NM_177966.5:p.Thr306_Tyr307fs/c.916_917insA | Microphthalmia With Limb Anomalies, not consistent with symptoms |
| *ZDHHC4* | chr7:6623063 |  | NM_001134387.1:c.496+2_496+3insT, | Not associated with human disease. Unlikely to cause a dominant disease due to monoallelic loss of function (pLI=5.3E-10) |
| *NFKB2* | chr10:104160444 |  | NM_001288724.1:p.Arg611*/c.1831C>T | Immunodeficiency, DAVID Syndrome (MIM #615577) |

**High effect –** likely loss of function due to premature stop codon, frameshift or splicing defect, or predicted as damaging by MetaSVM programme (<https://varsome.com>).

**Available databases –** gnomAD (<http://gnomad.broadinstitute.org/>), Ambry genetics database (<https://share.ambrygen.com>), in house database of >1000 Polish exomes

**pLI** – probability that monoallelic loss of function of a gene is pathogenic in human (<http://exac.broadinstitute.org>)
