# Supplementary material for: A Novel Monoallelic Nonsense Mutation in the NFKB2 Gene Does Not Cause a Clinical Manifestation
Source: Front Genet. 2019 Feb 26;10:140. doi: 10.3389/fgene.2019.00140 (PMC6399389; doi:10.3389/fgene.2019.00140)
Supplement: Supplementary file 5 [file Table_2.DOCX]

**S2 Table. Blood morphology.**

| **Subject** | **I.1** | **I.2** | **Normal range** |
| --- | --- | --- | --- |
| **White blood cells** |  |  |  |
| WBC (10^3^/μL) | 5.3 | 5.4 | 4.1 – 10.5 |
| LYM (%) | 35.7 | 40.0 | 16.0 – 43.3 |
| MON (%) | 5.6 | 5.2 | 2.8 – 10.2 |
| GRA (%) | 58.7 | 54.8 | 48.5 – 80.3 |
| LYM (10^3^/μL) | 1.90 | 2.10 | 1.00 – 3.10 |
| MON (10^3^/μL) | 0.20 | 0.20 | 0.10 – 0.70 |
| GRA (10^3^/μL) | 3.20 | 3.10 | 2.3 – 7.70 |
|  |  |  |  |
| **Red blood cells** |  |  |  |
| RBC (10^6^/μL) | 4.34 | **4.24** | 4.30 – 5.80 |
| HGB (g/dL) | 14.4 | 14.4 | 13.1 – 16.7 |
| HCT (%) | 38.4 | 38.5 | 39.9 – 51.0 |
| MCV (μm^3^) | 88 | 91 | 83 – 98 |
| MCH (pg) | **33.1** | **34.0** | 27.0 – 32.2 |
| MCHC (g/dL) | **37.4** | **37.5** | 31.8 – 33.7 |
| RDW (%) | 13.2 | 13.1 | 11.9 – 14.8 |
|  |  |  |  |
| **Platelets** |  |  |  |
| PLT (10^3^/μL) | 258 | 180 | 150 – 399 |
| MPV (μm^3^) | 8.6 | 9.8 | 6.8 – 10.1 |
| PCT (%) | 0.222 | 0.177 | 0.150 – 0.500 |
| PDW (%) | 11.7 | 15.5 | 11.0 – 18.0 |
