# Supplementary material for: A Novel Monoallelic Nonsense Mutation in the NFKB2 Gene Does Not Cause a Clinical Manifestation
Source: Front Genet. 2019 Feb 26;10:140. doi: 10.3389/fgene.2019.00140 (PMC6399389; doi:10.3389/fgene.2019.00140)
Supplement: Supplementary file 6 [file Table_3.DOCX]

**S3 Table. Lymphocytes T immunophenotyping.**

| **Subject** | **I.1** | **I.2** |
| --- | --- | --- |
| White blood cells (cells/μL) | 5300 | 5400 |
| Lymphocyte subpopulations |  |  |
| Total lymphocyte count (cells/μL) | 1892 | 2160 |
|  |  |  |
| Lymphocytes T subpopulations |  |  |
| CD3+ (cells/μL) | 1470 | 1348 |
| CD4+ (cells/μL) | 713 | 795 |
| Naive: CD4+CD45RA+CCR7+CD27+CD28+  CD127+CD279- (cells/μL) | 229 | 289 |
| Central memory: CD4+CD45RA-CCR7+CD27++CD28++ CD127++CD279- (cells/μL) | 102 | 131 |
| Effector memory: CD4+CD45RA-CCR7-CD27+CD28+ CD127+CD279+ (cells/μL) | 36 | 41 |
| TEMRA: CD4+CD45RA+CCR7-CD27-CD28+  CD127+CD279+- (cells/μL) | 18 | 5 |
|  |  |  |
| CD8+ (cells/μL) | 630 | 497 |
| Naive: CD8+CD45RA+CCR7+CD27+CD28+  CD127+CD279- (cells/μL) | 37 | 29 |
| Central memory: CD8+CD45RA-CCR7+CD27++CD28++ CD127++CD279- (cells/μL) | 7 | 12 |
| Effector memory: CD8+CD45RA-CCR7-CD27+CD28+ CD127+CD279+ (cells/μL) | 13 | 11 |
| TEMRA: CD8+CD45RA+CCR7-CD27-CD28+  CD127+CD279+- (cells/μL) | 48 | 54 |
